# DTL reconciliation repair

**DOI:** 10.1186/s12859-017-1463-9

**Published:** 2017-03-14

**Authors:** Weiyun Ma, Dmitriy Smirnov, Ran Libeskind-Hadas

**Affiliations:** 1 Department of Computer Science, Harvey Mudd College, Claremont, California USA; 2 Pomona College, Claremont, California USA

**Keywords:** DTL reconciliation, Phylogenetic reconciliation, Undated trees

## Abstract

**Background:**

Maximum parsimony phylogenetic tree reconciliation is an important technique for reconstructing the evolutionary histories of hosts and parasites, genes and species, and other interdependent pairs. Since the problem of finding temporally feasible maximum parsimony reconciliations is NP-complete, current methods use either exact algorithms with exponential worst-case running time or heuristics that do not guarantee optimal solutions.

**Results:**

We offer an efficient new approach that begins with a potentially infeasible maximum parsimony reconciliation and iteratively “repairs” it until it becomes temporally feasible.

**Conclusions:**

In a non-trivial number of cases, this approach finds solutions that are better than those found by the widely-used Jane heuristic.

## Background

Phylogenetic tree reconciliation is a fundamental technique for studying the evolution of pairs of entities such as gene families and species, parasites and their hosts, and species and their geographical habitats. The reconciliation problem takes as input two trees and the associations between their leaves and seeks to find a mapping between the trees that accounts for their incongruence. In the Duplication-Transfer-Loss (DTL) model, four types of events are considered: *speciation*, *duplication*, *transfer*, and *loss* [1–7].

**Fig. 1:**
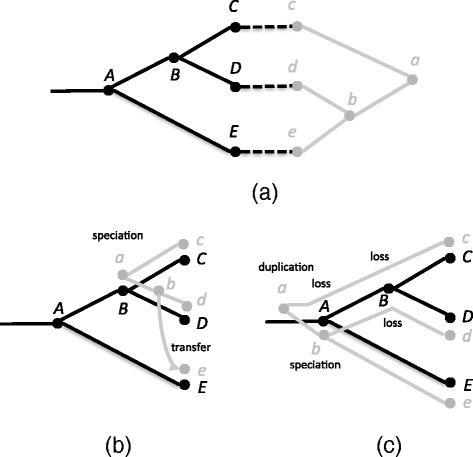
**a** A host tree in black and a parasite tree in gray with the leaf mapping shown with dotted lines. Two different reconciliations that are optimal for different event costs are shown in **b** and **c**

Reconciliation in the DTL model is typically performed using a maximum parsimony formulation where each event type has an assigned cost and the objective is to find a reconciliation of minimum total cost. Figure 1 a shows a small example of a host and parasite tree and their leaf associations. Figure 1 b and c show two different reconciliations of these trees with labels on the events. Speciation is generally considered a “null event” and given cost 0 while the other event types are given positive costs. For example if duplication, transfer, and loss each have cost 1, then the reconciliation in Fig. 1 b is optimal and incurs one speciation and one transfer, with total cost of 1. However, if duplication and loss have cost 1 and transfer has cost greater than 4, then the reconciliation in Fig. 1 c is optimal, incurring one speciation, one duplication, and three losses, with total cost of 4. Henceforth, we use the terms *optimal* and *maximum parsimony* interchangeably.

A host tree is said to be *dated* if the relative times of its internal nodes are known. For dated host trees, maximum parsimony reconciliations can be found in polynomial time [6, 8, 9]. However, accurately dating host trees is generally difficult [10], and estimated dates may be unreliable. Thus, much of the literature on DTL reconciliation assumes that the host tree is *undated*. Our work addresses the case of undated host trees.

**Fig. 2:**
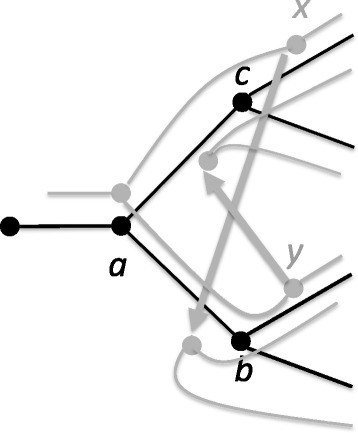
A fragment of a temporally infeasible reconciliation for a host tree (*black*) and parasite tree (*gray*). Node *x* transfers one child to the species edge from *a* to *b*, implying that *x* must occur before *b* and thus that *c* occurs before *b*. Node *y* transfers one child to the species edge from *a* to *c*, meaning that *y* must occur before *c* and thus that *b* occurs before *c*, contradicting the constraint that *c* occurs before *b*

In undated host trees, maximum parsimony reconciliations can be found in polynomial time using dynamic programming [1, 7, 9, 11], but these reconciliations may be *temporally infeasible* in the sense that there exists no ordering of the internal nodes that is consistent with the reconciliation. An example of a temporally infeasible reconciliation is illustrated in Fig. 2. Temporal infeasibility can be detected in polynomial time [11] but the problem of finding temporally feasible maximum parsimony reconciliations is NP-complete [7, 12]. Nonetheless, the dynamic programming solution provides a lower bound on the cost of a temporally feasible optimal solution.

In many applications, it is important that reconciliations be both temporally feasible and as close to optimal as possible [13]. Conclusions drawn from temporally infeasible or suboptimal solutions are dramatically weakened [14]. For example, in cophylogenetic studies of hosts and parasites, congruence of the host and parasite trees is assessed via randomization tests: The maximum parsimony cost of the original pair of trees is compared to the maximum parsimony costs for a sample of randomized versions of the data (e.g., randomization of the leaf-mapping or the parasite tree). The fraction of random samples whose cost is as good or better than the cost of the original pair of trees provides an empirical *p*-value for the null hypothesis that the trees are congruent by chance. Conclusions about the evolutionary histories of hosts and parasites depend, therefore, on accurate comparisons of the samples - meaning that they should all be temporally feasible and their costs should be as close to optimal as possible.

A number of algorithms and software tools have been developed to find temporally feasible maximum parsimony reconciliations in the DTL model. For example, TreeMap [15] uses an exact but exponential time algorithm. ILPEACE [14] and CoRe-ILP [16] find optimal solutions using integer linear programming, which also has exponential worst-case running time. In contrast, the widely-used Jane [17] tool uses a faster meta-heuristic that searches a portion of the large space of possible datings of the host tree and, for each one, finds the maximum parsimony reconciliation for that dating, resulting in a temporally feasible but not necessarily optimal solution.

The solutions found by Jane are often optimal, which is verified when the cost of Jane’s solution is equal to the lower bound found by the dynamic programming solution. However, in a substantial number of cases, the dynamic programming solution is not temporally feasible and there is a gap between its cost and the least cost solution found by the Jane heuristic. In such cases, it is desirable to find better temporally feasible solutions using another approach.

In this paper, we propose a new approach for finding temporally feasible reconciliations. This approach runs in polynomial time and, in a non-trivial number of cases (11% in our experiments using the Tree of Life dataset [5]), gives more parsimonious solutions than those found by Jane. Even relatively small improvements in the parsimony cost for a fraction of cases can have profound impact on analyses and conclusions based on tree reconciliation.

Our approach uses a combination of both existing and new algorithms. We use the efficient U-MPR dynamic programming algorithm [1] to find a maximum parsimony reconciliation. (Similar algorithms were proposed in [7, 9]. We note that our algorithm is self-contained and fully general and can be applied to reconciliations found by any algorithm). Next, we test that reconciliation for temporal feasibility using an algorithm similar to one proposed by [7]. If the reconciliation is temporally feasible then it is, necessarily, an optimal solution. If, however, the reconciliation is determined to be temporally infeasible, we apply an iterative “repair” process that successively modifies the reconciliation until it becomes feasible. This process terminates, is efficient, and has an upper-bound on the increase in the cost of the solution.

We note that seminal work by Tofigh et al. [7] explores repairing temporally infeasible reconciliations in the Duplication-Transfer model. They give an exact algorithm that runs in time exponential in the cost of the reconciliation. The differences between that work and ours is that our algorithm addresses the Duplication-Transfer-Loss model, our algorithm runs in polynomial time but is not exact (i.e., does not guarantee optimal solutions), and we compare our results to the prevailing heuristic tool and show that it performs better in a non-trivial fraction of cases. Indeed, Tofigh et al. [7] note that the exponential running time of their algorithm is not a concern because in a large analysis of synthetically generated data sets, the classical dynamic programming algorithm for maximum parsimony never constructed a temporally infeasible solution [18]. Subsequent work [19] corroborated this pattern in other synthetically generated data but found that in a large real data set from the Tree of Life [5], over 17% of maximum parsimony reconciliations were temporally infeasible.

In summary, in some cases existing heuristics do not find the least cost temporally feasible reconciliations and, in those cases, it is highly desirable to find lower cost reconciliations if possible. In this paper:

1. We show how a combination of existing and new algorithms can be used to efficiently find temporally feasible DTL reconciliations.
2. We provide experimental results that demonstrate that this approach finds better solutions than Jane in over 10% of cases in a large real dataset.
3. We provide a software package called *Cheeta* (www.cs.hmc.edu/~hadas/cheeta) that implements this algorithm and compares the results to those found by Jane.

## Preliminaries

We adopt definitions and notation from Bansal [1]. Let *T* be a rooted tree and denote the sets of nodes, edges, leaves, and internal nodes of *T* by *V*(*T*), *E*(*T*), *Le*(*T*), and *I*(*T*) respectively. Let *rt*(*T*) denote the root node of *T*, *pa*_*T*_(*v*) the parent of node *v*, *Ch*_*T*_(*v*) the set of children of *v*, and *T*(*v*) the maximal subtree of *T* rooted at *v*. Let *d*_*T*_(*x,y*) be the number of edges on the path from *x* to *y*. Let *x*≤_*T*_ *y* if *y* is a node on the path between *rt*(*T*) and *x* (inclusive) and *x*≥_*T*_ *y* if *x* is a node on the path between *rt*(*T*) and *y* (inclusive). Nodes *x* and *y* are said to be *incomparable* if neither *x*≤_*T*_ *y* nor *y*≤_*T*_ *x*. Let *lca*_*T*_(*x,y*) be the least common ancestor (LCA) of *x* and *y* in tree *T*; that is, *lca*_*T*_(*x,y*) is the node *z* furthest (with respect to *d*_*T*_) from the root such that *z*≥_*T*_ *x* and *z*≥_*T*_ *y*.

### DTL-scenarios and reconciliations

Next we give definitions from [1] leading to the definition of the maximum parsimony reconciliation problem.

#### **Definition 1**

(DTL-scenario [1]) A *DTL-scenario* for trees *G* and *S* is a seven-tuple $ (\mathcal {L}, \mathcal {M}, \Sigma, \Delta, \Theta, \Xi, \tau)$, where $\mathcal {L} \colon {Le}(G) \to {Le}(S)$ represents a leaf-mapping from *G* to *S*, $\mathcal {M} \colon V(G) \to V(S)$maps each node of *G* to a node of *S*, the sets *Σ*, *Δ*, and *Θ* partition *I*(*G*) into speciation, duplication, and transfer nodes respectively, *Ξ*is a subset of edges of *G* that represent transfer edges, and *τ*:*Θ*→*V*(*S*) specifies the recipient (or “landing site”) for each transfer event, subject to the following constraints: Constraint 1: If *g*∈*Le*(*G*), then $\mathcal {M}(g) = \mathcal {L}(g)$. Constraint 2: If *g*∈*I*(*G*) and *g*_*ℓ*_ and *g*_*r*_ denote the children of *g*, then,

1. $\mathcal {M}(g) \not <_{S} \mathcal {M}(g_{\ell })$ and $\mathcal {M}(g) \not <_{S} \mathcal {M}(g_{r})$,
2. At least one of $\mathcal {M}(g_{\ell })$ and $\mathcal {M}(g_{r})$ is a descendant of $\mathcal {M}(g)$.

Constraint 3: Given any edge (*g,g* ^′^)∈*E*(*G*), (*g,g* ^′^)∈*Ξ* if and only if $\mathcal {M}(g)$ and $\mathcal {M}(g')$ are incomparable. Constraint 4: If *g*∈*I*(*G*) and *g*_*ℓ*_ and *g*_*r*_ denote the children of *g*, then,

1. *g*∈*Σ* only if $\mathcal {M}(g) = lca_{S}(\mathcal {M}(g_{\ell }), \mathcal {M}(g_{r}))$ and $\mathcal {M}(g_{\ell })$ and $\mathcal {M}(g_{r})$ are incomparable,
2. *g*∈*Δ* only if $\mathcal {M}(g) \geq _{S} lca_{S}(\mathcal {M}(g_{\ell }), \mathcal {M}(g_{r}))$,
3. *g*∈*Θ* if and only if either (*g,g*_*ℓ*_)∈*Ξ* or (*g,g*_*r*_)∈*Ξ*.
4. If *g*∈*Θ* and (*g,g* ^′^)∈*Ξ*, then $\mathcal {M}(g)$ and *τ*(*g*) must be incomparable, and $\mathcal {M}(g')$ must be a descendant of *τ*(*g*), i.e., $\mathcal {M}(g') \leq _{S} \tau (g)$.

These four constraints ensure that (1) $\mathcal {M}$ extends $\mathcal {L}$, (2) $\mathcal {M}$ satisfies the temporal constraints from *S* and that each internal node in *G* is associated with at most one transfer event, (3) a transfer can only be to a non-ancestrally related node and (4) an internal node of *G* is designated with one of the four event types.

Note that while DTL-scenarios represent reconciliations, these reconciliation are not guaranteed to be temporally feasible. Next, losses are inferred from DTL scenarios according to the following definition from [1].

#### **Definition 2**

(Losses [1]) Given a DTL-scenario $\alpha = (\mathcal {L}, \mathcal {M}, \Sigma, \Delta, \Theta, \Xi, \tau)$ for *G* and *S*, let *g*∈*V*(*G*) and {*g*_*ℓ*_,*g*_*r*_}=*Ch*_*G*_(*g*). The number of *losses* *Loss*_*α*_(*g*) at node *g*, is defined to be:

- $(d_{S}(\mathcal {M}(g), \mathcal {M}(g_{\ell })) - 1) + (d_{S}(\mathcal {M}(g), \mathcal {M}(g_{r})) - 1)$, if *g*∈*Σ*,
- $d_{S}(\mathcal {M}(g), \mathcal {M}(g_{\ell })) + d_{S}(\mathcal {M}(g), \mathcal {M}(g_{r}))$, if *g*∈*Δ*, and
- $d_{S}(\mathcal {M}(g), \mathcal {M}(g_{r})) + d_{S}(\tau (g), \mathcal {M}(g_{\ell }))$ if (*g,g*_*ℓ*_)∈*Ξ*.

The total number of losses in the reconciliation corresponding to the DTL-scenario *α* is defined to be $Loss_{\alpha } = \sum _{g \in I(G)} Loss_{\alpha }(g)$.

Speciations are assumed to have zero cost. Duplications, transfers, and losses have positive costs denoted *C*_*Δ*_, *C*_*Θ*_, and *C*_*Λ*_, respectively.

#### **Definition 3**

(Reconciliation cost of a DTL-scenario [1]) Given a DTL-scenario $\alpha = (\mathcal {L}, \mathcal {M}, \Sigma, \Delta, \Theta, \Xi, \tau)$ for *G* and *S*, the *reconciliation cost* associated with *α* is given by *C*_*Δ*_·|*Δ*|+*C*_*Θ*_·|*Θ*|+*C*_*Λ*_·*Loss*_*α*_.

An instance of the maximum parsimony reconciliation problem comprises a gene tree *G*, a species tree *S*, a leaf mapping $\mathcal {L}: {Le}(G) \to {Le}(S)$, and positive costs *C*_*Δ*_, *C*_*Θ*_, and *C*_*Λ*_ for duplication, transfer, and loss events, respectively. A maximum parsimony reconciliation, henceforth denoted *MPR*, is a DTL-reconciliation of minimum total cost with respect to the given set of event costs.

A number of closely-related dynamic programming algorithms have been given for finding MPRs in undated trees [1,7,9]. Here, we use the U-MPR Algorithm from Bansal et al. [1] which has running time of *O*(|*G*||*S*|).

## The reconciliation repair algorithm

In this section, we describe a process for computing temporally feasible MPRs. The process begins by using the U-MPR algorithm [1] to find a most parsimonious DTL-scenario. Next, this scenario is tested for temporal feasibility using an algorithm similar to one described by Tofigh [11]. If the scenario is not temporally feasible, a particular gene node is selected and re-mapped to a species node higher up in the species tree, resulting in a new DTL-scenario. The process of choosing a gene node, remapping it, and testing the resulting scenario for feasibility is repeated until the scenario becomes temporally feasible. In this section, we describe the repair process, analyze it, and prove its correctness.

We determine if a DTL-scenario is temporally feasible using a *temporal feasibility graph* *F*=(*V,E*) constructed as follows:

1. *V*=*I*(*S*)∪*I*(*G*)∪{*ℓ*} where *I*(*S*) and *I*(*G*) represent the internal nodes of *S* and *G*, respectively, and *ℓ* is a single node representing all of the leaves of *S* and *G*.
2. For each pair of nodes *u,v*∈*V* such that *u* is the parent of *v* in either *S* or *G*, there is a directed edge (*u,v*)∈*E*.
3. For each *v*∈*V* such that its corresponding node in *S* or *G* is the parent of a leaf, there is an edge (*v*,*ℓ*)∈*E*.
4. For each gene node *g* associated with species node *s* in the DTL-scenario:
  - If the association is via a speciation event, *g* and *s* are identified (i.e., *g* is removed from the graph and all edges entering *g* are redirected to enter *s* and all nodes leaving *g* now leave *s*).
  - If the association is via a duplication event, we add the directed edges (*pa*_*S*_(*s*),*g*) (unless *s*=*rt*(*S*)) and (*g,s*).
  - If the association is via a transfer event with landing site *s* ^′^, we add the directed edges (*pa*_*S*_(*s*),*g*),(*g,s*),(*pa*_*S*_(*s* ^′^),*g*),(*g,s* ^′^).

A directed edge (*u,v*) represents the constraint that node *u* must have a date that comes before the date of *v*. Thus, the edges in 2 and 3 above enforce that ancestor nodes must have dates that come before their descendants. The edges in 4 enforce the relative dates of genes and the species with which they are associated. In particular, 4(c) ensures that the dates of takeoff and landing sites for transfers are contemporaneous. It is easily verified that there exists a dating of the tree in which all events are temporally consistent if and only if the temporal feasibility graph is acyclic. Moreover, if the graph is acyclic, a topological ordering of that graph gives a feasible dating for the species tree in the given DTL-scenario. (One difference between this test and the one in [11] is that we test that the reconciliation is temporally consistent given the takeoff and landing sites for each transfer event. The test in [11] does not specify the landing sites of transfer events and thus may determine that the scenario is feasible by moving landing sites to locations that are not consistent with any DTL-scenario).

If the DTL-scenario is found to be temporally infeasible due to a cycle in the temporal feasibility graph *F*, then the “repair” process identifies a gene node *g*, currently mapped to a species node $\mathcal {M}(g)$, such that *g* is on a cycle in *F* and such that no other gene node on a cycle in *F* is mapped to a descendant of $\mathcal {M}(g)$. In general, node *g* is not unique and the algorithm breaks ties arbitrarily.

**Fig. 3:**
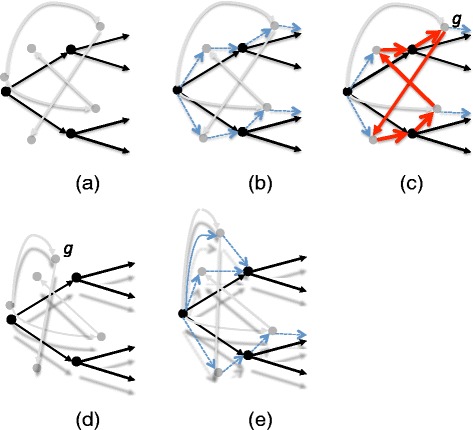
**a** The reconciliation from Fig. 2 with unimportant edges removed and edges directed from parent to children nodes. **b** The temporal feasibility graph for this reconciliation. **c** A cycle in the temporal feasibility graph with a minimal vertex *g* in the gene tree. **d** The modified reconciliation after vertex *g* is pulled up one level in the species tree. **e** The resulting temporal feasibility graph is acyclic and thus the resulting reconciliation is temporally feasible







Next, the mapping $\mathcal M$ is altered so that *g* is re-mapped (or “pulled up”) in the species tree by either moving to the parent of its current species node (if *g* is a duplication or transfer node) or to the edge above it (if *g* is a speciation node). The temporal feasibility graph is recomputed and this process is repeated until the temporal feasibility graph has no cycles, resulting in a temporally feasible DTL-scenario. Figure 3 illustrates a small example, and the process is described formally in Algorithms 1, 2, and 3 where Algorithm 1 is the main algorithm which invokes Algorithm 2 to select a gene node on a cycle and Algorithm 3 to move that gene node upward in the species tree.

### **Lemma 1**

Algorithm *1* terminates.

### *Proof*

We first prove that Algorithm 2 never returns a node *g*∈*I*(*G*) such that $\mathcal M(g) = rt(S)$. By way of contradiction, assume that Algorithm 2 returns such a node *g*. Then *g* is contained in some cycle *C* in the temporal feasibility graph *F*=(*V,E*). Because the for loop on line 2 of Algorithm 2 iterates over the internal nodes of *G* sorted by $\mathcal {M}(g)$ in post-order, any other node *g* ^′^∈*I*(*G*) (or the node that *g* ^′^ is identified with) contained in cycle *C*, if it exists, also has $\mathcal {M}(g') = rt(S)$. By construction of *F*, (*s,g* ^′^)∉*E* for any pair of nodes *s*∈*I*(*S*)∪{*ℓ*} and *g* ^′^∈*I*(*G*) such that $\mathcal {M}(g') = rt(S)$. Therefore cycle *C* does not contain any node *s*∈*I*(*S*)∪{*ℓ*} and consists only of nodes in the set $\{g' \in I(G) : \mathcal {M}(g') = rt(S)\}$. However, by construction of *F*, the subgraph induced by $\{g' \in I(G) : \mathcal {M}(g') = rt(S)\}$ is acyclic. Thus, we have a contradiction.

Let *h*_*S*_ denote the height of tree *S*. We now prove that throughout Algorithm 1, Algorithm 2 will return any given node *g*∈*I*(*G*) at most *h*_*S*_ times. If initially *g*∉*Σ*, then each time after *g* is returned by Algorithm 2, $\mathcal {M}(g)$ gets remapped to $pa_{S}(\mathcal {M}(g))$. If initially *g*∈*Σ*, the first time after *g* is returned by Algorithm 2, $\mathcal {M}(g)$ is not altered, but after every subsequent iteration the above claim applies. Since we have proven that Algorithm 2 will never return a node *g*∈*I*(*G*) such that $\mathcal M(g) = rt(S)$, Algorithm 2 returns any *g*∈*I*(*G*) at most *h*_*S*_ times.

It follows directly that the while loop in Algorithm 1 goes through at most |*I*(*G*)|*h*_*S*_ iterations. Therefore, Algorithm 1 terminates. □

### **Lemma 2**

Algorithm *3* returns a valid DTL-scenario.

### *Proof*

Let $\mathcal {M}'(\cdot)$ and *τ* ^′^(·) denote the updated mappings $\mathcal {M}(\cdot)$ and *τ*(·), respectively, when Algorithm 3 returns *α* at line 17. Let {*g*_*ℓ*_,*g*_*r*_}=*Ch*_*G*_(*g*).

We consider each constraint in Definition 1. Clearly, Constraint 1 holds throughout, since we never change $\mathcal M(g)$ for a leaf node *g*∈*Le*(*g*).

If *g* is initially a speciation node (*g*∈*Σ*), then by Constraint 4.1, $\mathcal M(g) = lca_{S}(\mathcal M(g_{\ell }), \mathcal M(g_{r}))$. When Algorithm 3 terminates, we have $\mathcal M'(g) = \mathcal M(g)$ and *g* is now a duplication node. We need only confirm that Constraints 2 and 4.2 hold. Indeed, $\mathcal M'(g) = \mathcal M(g) = lca_{S}(\mathcal M(g_{\ell }), \mathcal M(g_{r})) = lca_{S}(\mathcal M'(g_{\ell }), \mathcal M'(g_{r}))$, since $\mathcal M(g_{\ell }) = \mathcal M'(g_{\ell })$ and $\mathcal M(g_{r}) = \mathcal M'(g_{r})$. It follows trivially that Constraint 2 holds as well.

If *g* is initially a transfer node (*g*∈*Θ*), then since Constraints 2 and 4.3 hold at the beginning of the algorithm, exactly one of (*g,g*_*ℓ*_) and (*g,g*_*r*_) is in *Ξ*. Without loss of generality, suppose (*g,g*_*ℓ*_)∈*Ξ*. Then $\mathcal M(g) \ge _{S} \mathcal M(g_{r})$.

- If $\mathcal M'(g) >_{S} \tau (g)$, then when Algorithm 3 terminates, *g* is a duplication node and neither (*g,g*_*ℓ*_) nor (*g,g*_*r*_) is in *Ξ*. We need only confirm that Constraints 2, 3, 4.2 and 4.3 hold. Constraints 2 and 4.2 hold since $\mathcal M'(g) >_{S} \tau (g) \ge _{S}\mathcal M(g_{\ell }) = \mathcal M'(g_{\ell })$ and $\mathcal M'(g) = pa_{S}(\mathcal M(g)) >_{S} \mathcal M(g) \ge _{S} \mathcal M(g_{r}) = \mathcal M'(g_{r})$. It follows that Constraints 3 and 4.3 hold as well.
- Otherwise, since $\mathcal M'(g) = pa_{S}(\mathcal M(g)) >_{S} \mathcal M(g)$ and $\mathcal M(g)$ and *τ*(*g*) are incomparable, it must be the case that $\mathcal M'(g)$ and *τ*(*g*) are incomparable. When Algorithm 3 terminates, *g* remains a transfer node. We need only confirm that Constraints 2, 3, 4.3 and 4.4 still hold. Indeed, Constraint 4.3 holds since *Ξ* remains unchanged during Algorithm 3. Constraint 4.4 holds since $\mathcal M'(g)$ and *τ* ^′^(*g*)=*τ*(*g*) are incomparable and $\mathcal M'(g_{\ell }) = \mathcal M(g_{\ell }) \le _{S} \tau (g) = \tau '(g) $. It follows that $\mathcal M'(g)$ and $\mathcal M'(g_{\ell })$ are also incomparable, so Constraint 3 also holds. Moreover, $\mathcal M'(g_{r}) = \mathcal M(g_{r}) \le _{S} \mathcal M(g) <_{S} pa_{S}(\mathcal M(g)) = \mathcal M'(g)$, so Constraint 2 holds.

If *g* is initially a duplication node (*g*∈*Δ*), then by Constraint 4.2, $\mathcal M(g) \ge _{S} lca_{S}(\mathcal M(g_{\ell }), \mathcal M(g_{r}))$. When Algorithm 3 terminates, *g* remains a duplication node. We need only confirm that Constraints 2 and 4.2 still hold. Indeed, $\mathcal M' (g) = pa_{S}(\mathcal M(g)) >_{S} \mathcal M(g) \ge _{S} lca_{S}(\mathcal M(g_{\ell }), \mathcal M(g_{r})) = lca_{S}(\mathcal M'(g_{\ell }), \mathcal M'(g_{r}))$. It follows trivially that Constraint 2 holds as well. Therefore, Algorithm 3 returns a valid DTL-scenario. □

### **Lemma 3**

Algorithm *1* returns a valid DTL-scenario.

### *Proof*

The algorithm takes as input a valid DTL-scenario, and by Lemma 2, at every iteration of the while loop, a valid DTL-scenario is returned. Therefore, by induction, Algorithm 1 returns a valid DTL-scenario. □

### **Theorem 1**

Algorithm *1* returns a temporally consistent DTL-scenario for *G* and *S*.

### *Proof*

By Lemma 1 and Lemma 2, we know that at some point Algorithm 2 returns *null* with input *G*, *S* and a valid DTL-scenario *α*, which means that there does not exist any cycle containing any *g*∈*I*(*G*) in the temporal feasibility graph *F* corresponding to *α*. Moreover, by construction of *F*, the subgraph induced by *I*(*S*)∪{*ℓ*} is acyclic. Therefore *F* is acyclic. It follows that *α* is temporally consistent. □

Let *h*_*S*_ and *h*_*G*_ denote the heights of trees *S* and *G*, respectively. We now bound the running time of Algorithm 1 and the increase in the number of events introduced.

### **Lemma 4**

The worst-case time complexity of Algorithm *3* and Algorithm *2* is *O*(1) and *O*(|*G*|^2^+|*G*||*S*|), respectively.

### *Proof*

The time complexity of Algorithm 3 is trivially *O*(1). For Algorithm 2, note that the size of the temporal feasibility graph *F* corresponding to any *α* is in *O*(|*G*|+|*S*|). Therefore it takes *O*(|*F*|)=*O*(|*G*|+|*S*|) time to construct *F* and to check if there exists a cycle in *F* containing any given node *g* using depth-first search. Also, since the for loop goes through *O*(|*G*|) iterations, the total time complexity of Algorithm 2 is *O*(|*G*|^2^+|*G*||*S*|). □

### **Theorem 2**

The worst-case time complexity of Algorithm *1* is *O*(|*G*|^3^ *h*_*S*_+|*G*|^2^|*S*|*h*_*S*_).

### *Proof*

There is a single invocation of the U-MPR algorithm whose worst-case running time is *O*(|*G*||*S*|). Since Algorithm 1 goes through at most |*I*(*G*)|*h*_*S*_∈*O*(|*G*|*h*_*S*_) iterations and it calls Algorithm 3 and Algorithm 2 once respectively at each iteration, from Lemma 4 it follows that the total time complexity of Algorithm 1 is *O*(|*G*|^3^ *h*_*S*_+|*G*|^2^|*S*|*h*_*S*_). □

### **Lemma 5**

Let *α* denote the initial DTL-scenario and let *Θ* represent the transfer nodes in *α*. If Algorithm *1* invokes Algorithm *2*, which in turn returns *g*∈*V*(*G*), then there must exist some *g* ^′^∈*Θ* such that *g*≥_*G*_ *g* ^′^.

### *Proof*

By the definition of Algorithm 2, *g* is on a cycle in the graph *F*. Denote the arcs of *F*, except for those defined by step 4(c) in the construction, as white arcs and let the arcs defined by step 4(c) be black arcs. Note that, by construction, there can be no cycles that involve exclusively white arcs and thus the cycle *C* detected in line 3 of Algorithm 4 necessarily involves at least one black arc. Consider the subpath of *C* from *g* to the first black arc *e* on *C*. By construction of *F*, arc *e* was introduced by a transfer event *g* ^′^∈*Θ* and that transfer event is reachable by white arcs from *g* and thus *g*≥_*G*_ *g* ^′^. □

### **Theorem 3**

Algorithm *1* introduces at most *kh*_*G*_ duplication events and *kh*_*G*_ *h*_*S*_ loss events, where *k* is the number of transfer events in the initial MPR *α*.

### *Proof*

We first prove that Algorithm 1 introduces at most *kh*_*G*_ duplication events. A new duplication event can be introduced at most once for each node *g*∈*I*(*G*). By Lemma 5, we know that only a transfer node and its ancestors may be modified by our algorithm. Assume that, in the worst case, every transfer node and every ancestor of a transfer node is modified and that the sets of ancestors are disjoint for each transfer node. The number of ancestors for a transfer node is strictly less than the height of the tree *h*_*G*_, and we have a total of *k* transfer nodes. Therefore, no more than *kh*_*G*_ duplication events are introduced.

We now prove that Algorithm 1 introduces at most *kh*_*G*_ *h*_*S*_ loss events. We may introduce a series of loss events each time we make *g* a new duplication node. Based on the definition of the number of losses, *Loss*_*α*_(*g*)≤*h*_*S*_. Therefore, no more than *kh*_*G*_ *h*_*S*_ loss events are introduced. □

## Results

To demonstrate the utility of our approach, we ran our algorithm on a dataset comprising 4848 parasite trees for a host tree comprising 100 (predominantly prokaryotic) species from the Tree of Life [5]. We used DTL values of 2, 3, and 1, respectively. In this dataset 17.4*%* of MPRs found by the U-MPR algorithm were temporally infeasible. In order to compare the solutions found by our algorithm to those found by Jane tool, we selected the first 100 of the 4848 parasite trees for comparison. (We do not compare performance to TreeMap or the ILP approaches since their exponential worst-case running times make them viable only for very small datasets).

In general, there may be multiple MPRs for a given DTL instance. Our implementation of the U-MPR algorithm can find all MPRs and we chose the first 10 and repaired all of them, if necessary, and reported the best score. Jane uses a genetic algorithm that maintains a population of *T* candidate datings for the host tree and runs for *P* iterations. We used *T*=30 and *P*=30 in these experiments.

In 27% of the cases, our algorithm found reconciliations with lower costs than those found by Jane: 16% that were temporally feasible and required no repair and 11% that were not temporally feasible and required repair. On average, in these cases, the repaired reconciliations had costs that were 5.5*%* lower than Jane’s costs. In the remaining 73% of cases, Jane performed at least as well as our algorithm. However, given that Jane is a de facto standard in many cophylogenetic studies, it is notable that better solutions can be obtained by our relatively simple and fast algorithm for a non-trivial fraction of cases.

Our code is available in the Cheeta package (www.cs.hmc.edu/~hadas/cheeta) which runs both Jane and our repair algorithm and reports the best solution found (from Jane, from U-MPR with no repair necessary, or from U-MPR with our repair algorithm).

## Conclusions

In this work we have described a new approach for finding temporally feasible reconciliations in the DTL model. This algorithm is efficient and, in a significant number of cases, finds solutions that are better than those found by the widely used Jane heuristic. In those cases, the results from our heuristic should be used instead of the results from Jane in order to draw more robust conclusions.

The “repair approach” described here has the desirable property that it begins with a reconciliation whose cost is a lower bound on that of a temporally feasible optimal solution. While we derive a bound on the increase in cost due to successive repair steps, this bound is quite large. Future work is needed to determine if this bound is tight or can be improved. Additionally, there may be other ways to repair temporally infeasible reconciliations that perform even better than the one described here. Finally, it is possible that this approach may lead to approximation algorithms or schemes for the DTL MPR problem.
